# 

**Published:** 1892-04

**Authors:** 


### HALL'S JOURNAL OF HEALTH

TRUTH DEMANDS NO SACRIFICE; ERROR CAN MAKE NONE.

Vol. 39. APRIL, 1892. No. 4.

### SPECIAL TO NEWS DEALERS.

We extract the following notices from the March number of the *Book of News Dealers*, published in San Francisco, Cal.:

HALL'S JOURNAL OF HEALTH is as familiar as a household word to dealer and reader all over the country. It retails for ten cents, and is always readable.—*News Dealers', Publishers' and Stationers' Bulletin.*

I would like to know what that household word is. A ten-cent paper costing a dealer eight cents, plus postage, has a very slim news-stand circulation these days, and that's what is the matter with HALL'S JOURNAL OF HEALTH.

In answer to the above, permit us to say that news dealers, wherever located, are permitted to send their orders directly to this office. All such orders will be filled and delivered to them by mail at twenty-five per cent. discount from the published rate, thus giving them an ample margin of profit. What, then, is the matter with

HALL'S JOURNAL OF HEALTH ?

### LITERARY.

EDITOR HALL'S JOURNAL OF HEALTH:

In your March issue you endorse HERBA VITA as a corrective and cure of many bodily derangements. What practical knowledge have you of its efficacy?

Respect., etc.

Scranton, March 9, 1892. THOS. L. OSGOOD.

We endorse HERBA VITA,—

1st.—Because we have known for a number of years the proprietors and place confidence in them, and what they say.

2nd.—For the past four years we have used with great satisfaction this remedy in our family, consisting of ourself and wife, wife's mother, two female servants and seven children, between the ages of 8 months and 13 years.

In fact, HERBA VITA has come to be almost our only *family doctor* for all ordinary complaints at a very considerable saving of fees and worriment, and so well and completely does it do its work, that we have seldom had occasion to resort to any other mode of treatment. [ED. MED. DEPT.]

THE MEDITERRANEAN SHORES OF AMERICA; or, THE CLIMATIC, PHYSICAL AND METEOROLOGICAL CONDITIONS OF SOUTHERN CALIFORNIA. By P. C. Remondino, M. D., Member of the State Board of Health of California, etc., etc. Illustrated with forty-five engravings and two double page maps. In one handsome, royal octavo volume, 176 pages. Extra cloth, price $1.25, net; cheaper edition, bound in paper, price, 75 cents, net. The F. A. Davis Co., Publishers, 1231 Filbert St., Philadelphia.

This is a serious and conscientious treatise on the climate of Southern California and its meteorological results, and contrasts most favorably with numerous hand-books which have, from time to time, been presented to the public, with the object of leading the chronic invalid, and especially the unfortunate consumptive to a genial home for the recuperation of his lost health and strength. No detail that in any way or to any extent bears upon the physical geography of this region of America, has escaped the author's notice or been left without its due weight and consideration in what might fitly be called his solemn effort to help suffering humanity and chiefly a large and constantly increasing class thereof—we mean consumptives. It is therefore wholly to the systematic, the scientific manner in which this little book has pursued and exhausted its subject, in which it has, apparently, reconciled the inconsistent and contradictory information which beset the path of the author in his humane and noble effort to solve the problem of medical climatology in its relations to the etiology of phthisis, that this work owes its characteristic feature of faithfulness and thoroughness, and on this account may, with safety and profit, be consulted, and even digested.

HUMANITY AND HEALTH.

This new candidate for popular favor has reached a second issue. Its projector and editor is the well and favorably known Doctor Jennings, a lady of indomitable will and untiring perseverance in all good works, as evinced in launching alone and almost unaided, a journal whose objects are explained by its title, and ably borne out in its pages.

We wish our new contemporary a success equal to its merits and that will be all that can be reasonably desired.

FIFTH AND SIXTH ANNUAL REPORTS OF THE STATE BOARD OF HEALTH AND VITAL STATISTICS OF THE COMMONWEALTH OF PENNSYLVANIA. 1474 pp.

These exhaustive reports show that the Keystone State is fairly in the lead in all matters which concern the health and sanitary well being of its citizens. The causes and prevention of many of the more prevalent diseases are treated of in a most comprehensive and instructive manner, and its WEATHER REPORTS and Vital Statistics show great thoroughness and painstaking in their computation.

THE AMERICAN SOCIETY FOR THE PREVENTION OF CRUELTY TO ANIMALS, 26th Annual Report.

Should any one question the value of the humanitarianism of this Society, let him peruse this Report, and be thoroughly convinced that human brutes are crueller by far than their dumb submissive superiors.

Our thanks are due to the several authors and compilers of the following publications:

Transactions of the American Octological Society, Vol. 5. Part 1, with a complete Alphabetical Index of Octological Literature, from July 1, 1890 to July 1, 1891. 177 pp. Published by the Society.

This, to the practical octologist, will be found to be of great value.

Notes on Beauty, Vigor and Development. Fowler & Wells Co. Price, 10 cents.

A very excellent work treating largely of physical exercise, with illustrations.

Presbyterian Eye, Ear, and Throat Charity Hospital. Fourteenth Annual Report. Baltimore, Md.

A most worthy charity.

Notes on General versus Local Treatment of Catarrhal Inflammations of the Upper Air Tract. By Beverly Robinson, M. D., New York.

Indications for Cototomy. By Professor Charles B. Kelsey, M. D., Detroit, Mich.

Tuberculin: The value and limitation of its use in consumption. By Professor Charles Denison, A. M., M. D., Denver, Col.

Empiricism.—Rational Practice—Practice under Guidance of Law. By Charles S. Mack, M. D., Ann Arbor, Mich.

